# Supplementary material for: Phylogeny and Taxonomic Revision of the Genus Melanosciadium (Apiaceae), Based on Plastid Genomes and Morphological Evidence
Source: Plants (Basel). 2024 Mar 21;13(6):907. doi: 10.3390/plants13060907 (PMC10974901; doi:10.3390/plants13060907)
Supplement: Supplementary file 1 [file plants-13-00907-s001.zip › Table S1.pdf]

Table S1. Sources of fruits of these species.

Species	Locality	Voucher
Melanosciadium pimpinelloideum	Nanchuan District, Chongqing City, China	JQP20091502
Melanosciadium jinzhaiensis	Jinzhai County, Anhui Province, China	JQP18102302
Melanosciadium bipinnatum	Wenchuan County, Sichuan Province, China	JQP20092602
Ligusticum angelicifolium	Dali City, Yunnan Province, China	JQP19091801
Angelica tsinlingensis	Huayin City, Shaanxi Province, China	JQP19101801-1
