# Supplementary material for: Phylogeny and Taxonomic Revision of the Genus Melanosciadium (Apiaceae), Based on Plastid Genomes and Morphological Evidence
Source: Plants (Basel). 2024 Mar 21;13(6):907. doi: 10.3390/plants13060907 (PMC10974901; doi:10.3390/plants13060907)
Supplement: Supplementary file 1 [file plants-13-00907-s001.zip › Table S5.pdf]

Table S5. List of species in this study with their GenBank accession number for 85 plastid genome sequences obtained from National Center for Biotechnology Information (NCBI).

Clade name	Genus name	Species name	GenBank accession numbers
		Saniculoideae	
	Sanicula	*Sanicula rubriflora*	MW690208
		Sanicula orthacantha	MK293943
		Sanicula lamelligera	MT561031
		Sanicula chinensis	MK208987
		Apioideae	
Chamaesium Clade	*Chamaesium*	*Chamaesium delavayi*	MN119367
		Chamaesium thalictrifolium	MN119372
		Chamaesium spatuliferum	MN119371
		Chamaesium jiulongense	MN119368
Bupleureae	*Bupleurum*	*Bupleurum smithii*	MN854382
		Bupleurum chinense	MN337347
		Bupleurum yinchowense	MT075711
		Bupleurum bicaule	MT534603
		Bupleurum angustissimum	MT534602
Pleurospermeae	*Pleurospermum*	*Pleurospermum camtschaticum*	KU041142
		Pleurospermum decurrens	OP425133
		Pleurospermum szechenyii	OP425139
		Pleurospermum franchetianum	MW147498
Physospermopsis	*Tongoloa*	*Tongoloa silaifolia*	MN218688
Clade	*Hansenia*	*Hansenia weberbaueriana*	KX808491
		Hansenia forrestii	MG197741
		Hansenia forbesii	KX808492
Komarovia Clade	*Chuanminshen*	*Chuanminshen violaceum*	KU921430
	Changium	*Changium smyrnioides*	MN092718
Oenantheae	*Cryptotaenia*	*Cryptotaenia japonica*	MK629764
	Cicuta	*Cicuta virosa*	KX352466
	Oenanthe	*Oenanthe javanica*	MK303392
		Oenanthe linearis	MT561035
	Trachyspermum	*Trachyspermum ammi*	MN746303
Scandicinae	*Anthriscus*	*Anthriscus sylvestris*	MT561042
		Anthriscus cerefolium	GU456628
	Ferula	*Ferula sinkiangensis*	MW411057
	Daucus	*Daucus carota*	DQ898156
	Cuminum	*Cuminum cyminum*	MN901636
Acronema Clade	*Meeboldia*	*Meeboldia yunnanensis*	MK993275
		Meeboldia delavayi	MT843765
		Meeboldia microloba	MT843766
	Pternopetalum	*Pternopetalum vulgare*	MT561032
		Pternopetalum davidii	MW316663

	Ostericum	*Ostericum huadongense*	MW436385
		Ostericum grosseserratum	MW436384
		Ostericum citriodorum	MT501096
		Ostericum palustre	MN970215
		Ostericum muliense	OM281949
		Ostericum scaberulum	OM281950
		Ostericum maximowiczii	OM281948
Apieae	*Apium*	*Apium graveolens*	MK036045
	Petroselinum	*Petroselinum crispum*	HM596073
	Foeniculum	*Foeniculum vulgare*	KR011054
	Anethum	*Anethum graveolens*	KR011055
Hymenidium Clade	*Ligusticum*	*Ligusticum pteridophyllum*	MT409617
		Ligusticum tenuissimum	KT963039
		Ligusticum jeholense	MT561037
		Ligusticum chuanxiong	KX594382
		Ligusticum sinense	MH260704
Coriandreae	*Coriandrum*	*Coriandrum sativum*	KR002656
Pimpinelleae	*Nothosmyrnium*	*Nothosmyrnium japonicum*	MT561036
Tordylieae	*Heracleum*	*Heracleum yunnanense*	MN365275
		Heracleum candicans	MK522402
		Heracleum yungningense	MN893285
		Heracleum moellendorffii	MK210561
	Semenovia	*Semenovia transiliensis*	MK333395
		Semenovia thomsonii	MW371294
		Semenovia gyirongensis	MK757488
	Tordyliopsis	*Tordyliopsis brunonis*	MW741883
Selineae	*Ligusticopsis*	*Ligusticopsis acaulis*	ON359911
		Ligusticopsis brachyloba	MZ491174
		Ligusticopsis capillacea	MT409612
		Ligusticopsis hispida	MT409614
		Ligusticopsis involucrata	MT409615
		Ligusticopsis likiangensis	MT409616
		Ligusticopsis miyiensis	OP850831
		Ligusticopsis modesta	OL547615
		Ligusticopsis rechingeriana	MZ491175
		Ligusticopsis scapiformis	MT409618
		Ligusticopsis wallichiana	OL547616
	Seseli	*Seseli montanum*	KM035851
	Peucedanum	*Peucedanum praeruptorum*	MN016968
		Peucedanum japonicum	KU866530
		Peucedanum terebinthaceum	MT671397
		Peucedanum hakuunense	OL362112
		Peucedanum chujaense	MT233391
	Libanotis	*Libanotis buchtormensis*	MZ707534

	Saposhnikovia	*Saposhnikovia divaricata*	MN539269
	Ledebouriella	*Ledebouriella seseloides*	KT153021
	Cnidium	*Cnidium monnieri*	MT561041
	Melanosciadium	*Melanosciadium bipinnatum*	MG719855
	Angelica	*Angelica apaensis*	MT921966
		Angelica biserrata	MT921960
		Angelica cartilaginomarginata	MT561045
		Angelica dahurica var. *formosana*	MT921971
		Angelica gigas	MH793272
		Angelica kangdingensis	MT921965
		Angelica keiskei	MW125613
		Angelica laxifoliata	MT921969
		Angelica morii	MT921982
		Angelica nitida	MF594405
		Angelica porphyrocaulis	MT921973
		Angelica pseudoselinum	MT921968
		Angelica sylvestris	MN275034
		Angelica valida	MT921963
