# Supplementary figures and images for: Phylogeny and Taxonomic Revision of the Genus Melanosciadium (Apiaceae), Based on Plastid Genomes and Morphological Evidence
Source: Plants (Basel). 2024 Mar 21;13(6):907. doi: 10.3390/plants13060907 (PMC10974901; doi:10.3390/plants13060907)

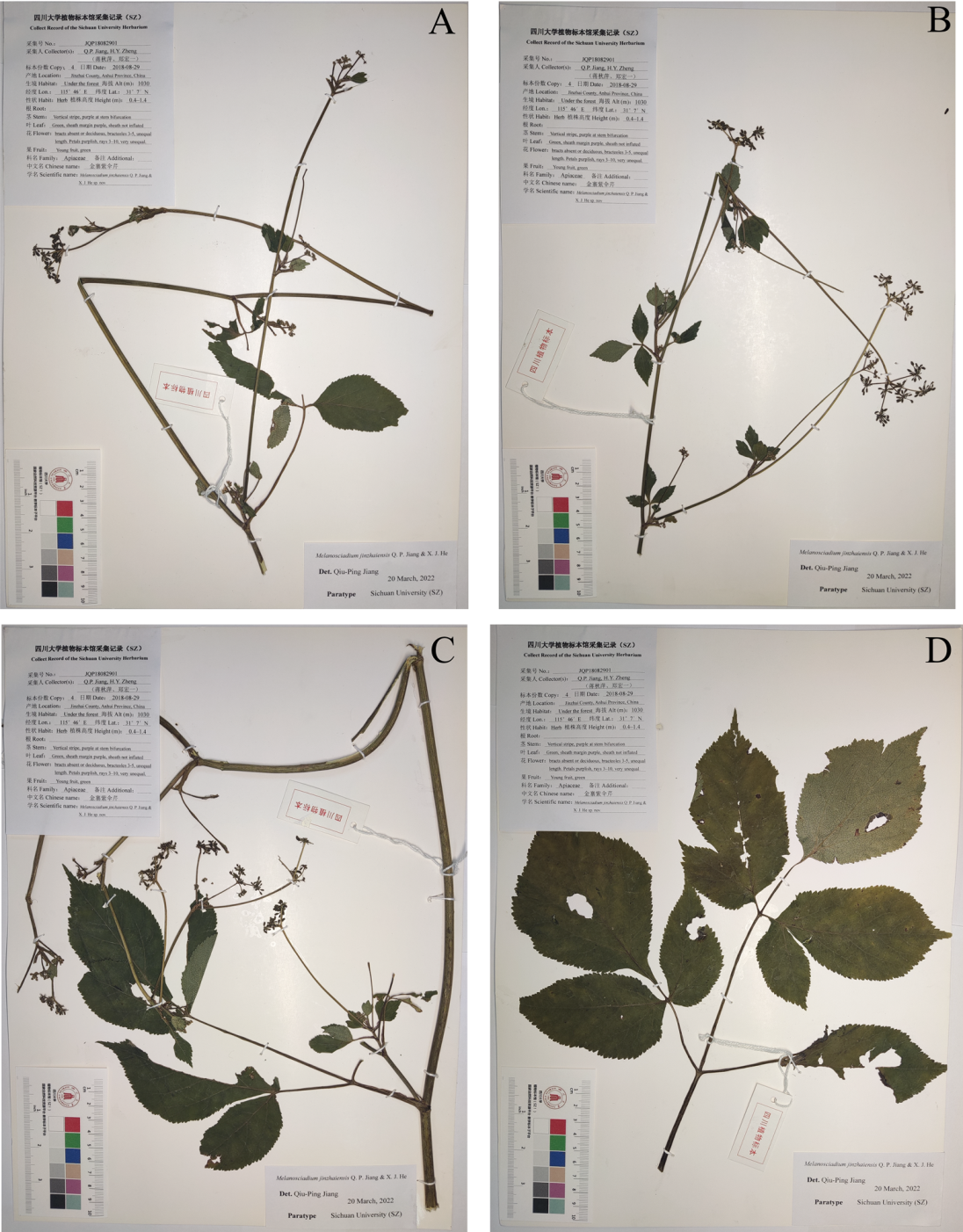

Supplement: Supplementary file 1 [file plants-13-00907-s001.zip › Figure S1.png]
